# Identification of a novel post-translational modification in *Plasmodium falciparum*: protein sumoylation in different cellular compartments

**DOI:** 10.1111/j.1462-5822.2008.01183.x

**Published:** 2008-10

**Authors:** Neha Issar, Emeric Roux, Denise Mattei, Artur Scherf

**Affiliations:** Institut Pasteur- CNRS URA 2581, Biology of Host-Parasite Interactions UnitF75724, Paris, France.

## Abstract

SUMO (Small Ubiquitin-like MOdifier) conjugation is a post-translational modification implicated in a variety of cellular functions including transcriptional regulation, nuclear location and signal transduction. Sumoylation, although conserved and vital in eukaryotes, has not been studied in malaria parasites. Here, we identify SUMO conjugation of blood stage parasites of *Plasmodium falciparum.* Antibodies raised against synthetic peptides of the plasmodial SUMO orthologue PfSUMO, a 100-amino-acid protein, reacted with distinctive subcellular compartments of the parasitized erythrocyte during blood stage development. Anti-PfSUMO stains the nucleus and parasite cytoplasm. We also found antibody reactivity in the host cell cytoplasm with the parasite-derived structures called Maurer's clefts. Anti-PfSUMO reacts in Western blot with a number of blood stage proteins ranging from approximately 40–250 kDa. Parasites expressing FLAG-tagged PfSUMO gave similar results in Immunofluorescence assay and Western blots. In addition, we show that anti-PfSUMO identified PfSir2, a telomere-associated nuclear protein involved in *var* gene silencing, as a target for sumoylation. Furthermore, LC-MS/MS analysis of a two-step immunoprecipitation (IP) with anti-FLAG and anti-PfSUMO antibodies reveals a number of putative *P. falciparum* sumoylated proteins. Our results imply that SUMO conjugation has an essential function in a number of different biological processes in *P. falciparum.*

## Introduction

Gene expression in eukaryotes is executed by a relatively small number of regulators, the dynamic prospects of which are dramatically increased by post-translational modifications. Reversible post-translational modifications of proteins, such as phosphorylation, acetylation, methylation and ubiquitination have critical roles in many cellular processes owing to their ability to cause rapid changes in functions of pre-existing proteins and subcellular structures. Covalent attachment of one protein to another, resulting in altered localization and/or function, is one of the best-studied post-translational modifications with respect to prevalence in eukaryotes. Among these, ubiquitination is the most studied; however, other ubiquitin-like proteins (Ubls), such as SUMO, Nedd 8 and ISGN15, are gaining increased attention (Jentsch and Pyrowolakis, 2000).

*Plasmodium falciparum*, the most virulent of the protozoan parasites infecting humans, causes a medically and socio-economically debilitating form of malaria. Although components of the ubiquitination pathway and related ubiquitin-like modification, such as neddylation has been recently reported in malaria parasites (Artavanis-Tsakonas *et al*., 2006) protein sumoylation in apicomplexan parasites has not been investigated. This, despite the fact that SUMO is emerging as a flexible modifier for a large number of proteins in many different pathways with diverse consequences and targets (Seeler and Dejean, 2003). SUMO is structurally related to ubiquitin (Bayer *et al*., 1998), is of similar size and is also ligated to lysine residues within its target proteins. This happens via a cascade of activation, conjugation and ligation steps, to produce an isopeptide bond between C-terminal glycine of SUMO and a specific lysine (within a short consensus sumoylation motif) of the target protein (Johnson, 2004). However, unlike ubiquitin it does not mark the protein for degradation, instead it modifies its substrate's interaction with partner proteins. In contrast to the single SUMO found in *Saccharomyces cerevisiae, Drosophila melanogaster* and *Caenorhabditis elegans*, higher eukaryotes express multiple SUMOs, with up to eight members in *Arabidopsis thaliana* and three in humans, namely SUMO1, SUMO2 and SUMO3. Mature SUMO2 and SUMO3 are nearly identical (∼95% identity) but differ substantially from SUMO1 (∼50% identity).

The complex life cycle of the human malaria parasite *P. falciparum*, divided between insect vector and human host, necessitates dynamic protein expression and gene regulation to exploit efficaciously the different host environments. Clearly the parasite requires a dynamic protein repertoire and a ubiquitous control of gene expression to achieve such complex tasks. This has been generally evidenced by reports of considerable levels of gene expression regulation throughout the *P. falciparum* life cycle (Florens *et al*., 2002; Lasonder *et al*., 2002). Furthermore, the scarcity of plasmodial transcription regulators identified (Coulson *et al*., 2004) indicates alternative modes of control. A good example of a prominent role of reversible post-translational modifications in gene regulation comes from the study of histone marks and the telomeric chromatin-associated PfSir2 protein in antigenic variation of *P. falciparum* (Freitas-Junior *et al*., 2005; Chookajorn *et al*. 2007; Cui *et al*., 2007; Lopez-Rubio *et al*., 2007). Here we demonstrate the presence of another post-translational modification in malaria parasites called sumoylation. We report data that imply a crucial role of SUMO-conjugated proteins in different processes during *P. falciparum* blood stage development.

## Results

### Identification of *P. falciparum* SUMO1/SMT3 homologue and sumoylation pathway components

**Fig. 1:**
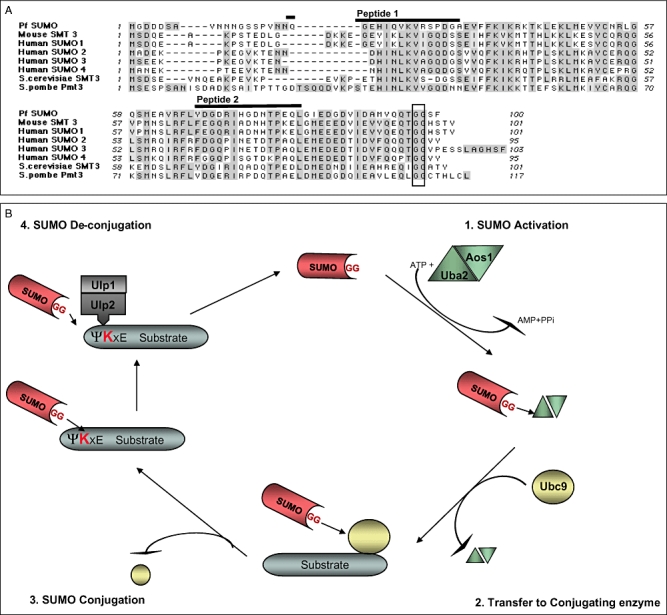
PfSUMO alignment with orthologous proteins. CLUSTAL W algorithm was used to ascertain PfSUMO homology to known SUMO orthologues. A. Multiple alignments between Mouse SMT3, Human SUMO1, 2 and 3, *S. cerevisiae* SMT3 and *S. pombe* Pmt3 was done. Dark shading represents residues identical in all sequences. Light shading, residues similar between respective sequences. Box, conserved di-glycine motif. Dashes, spaces inserted to achieve optimal match. Bold line above Pf sequence, most antigenic residues used to generate anti-peptide antibodies. B. Schematic of *P. falciparum* sumoylation pathway. *P. falciparum* orthologues for all members of the sumoylation pathway have been identified. The C-terminus of a mature SUMO (PFE0285c) containing the glycine–glycine motif, is first activated by activating enzyme E1, a dimer of Uba 2 (PFL1790w) and Aos1 (PF11-0457), linking it by a thioester to a cysteine residue in Uba2. Activated SUMO is then transferred to a cysteine residue of E2, a SUMO-specific conjugating enzyme Ubc9 (PFI0740c). In conjugation with SUMO ligases, Ubc9 conjugates SUMO to various substrate proteins. Target proteins contain SUMO motifs, whereby the SUMO binds to a specific lysine residue. Sumoylated substrates undergo de-conjugation via specific SUMO proteases, Ulp1(PFL1635w) and Ulp 2 (PFD 0970c). Another *P. falciparum* sumoylation pathway developed by H. Ginsburg can be found: http://sites.huji.ac.il/malaria/maps/SUMOylation.html

Growing evidence of the presence and role of ubiquitin-like modifiers in gene regulation in model eukaryotic systems led us to search for putative Ubls in *P. falciparum*. Amino acid sequence comparisons revealed a putative Ubl catalogued with gene ID PFE0285c (previously MAL5P1.58) in PlasmoDB as having the best score. Based on the best-hit score we have hereby called it PfSUMO. Multiple alignments with other previously characterized SUMO orthologues show that PfSUMO is 46% identical to *S. cerevisiae* SMT3, 40% identical to Human SUMO1, Mouse SMT3 and *S. pombe* Pmt3 and 47% and 43% to Human SUMO2 and 3 respectively (Fig. 1A). To exclude the possibility of obtaining a protein involved in ubiquitination or some other Ubls instead, we carried out similar alignment(s) with known yeast and human ubiquitin, revealing only about 11% and 7% identity, respectively, to PfSUMO (Fig. S1). Furthermore, PfSUMO lacks the critical Lys48 and Lys63 residue of ubiquitin, required for the generation of ubiquitin polymers and instead seems to possess the most prominent feature of SUMO1, a long N-terminus, which is believed to be highly flexible and protruding from the core of the protein (Bayer *et al*., 1998). However, the two C-terminal Gly residues required for isopeptide bond formation are conserved between all sequences. These bioinformatic analyses and observations together suggest the protein to be of the SUMO family. Interestingly, though it shares slightly more identity to SUMO2, other features such as high similarity score with *S. cerevisiae* SMT3 and apparent structural features such as the long N-terminal point it to be more similar to SUMO1.

The complete characterization of a protein involved in a crucial process necessitates deciphering and establishing its entire pathway. We thus carried out homology search for enzymes involved in the SUMO pathway for yeast and humans against the *P. falciparum* database. *P. falciparum* orthologues of all members of the pathway have been identified and the proposed pathway components are represented (Fig. 1B). Like its most widely known counterpart SMT3, SUMO1 is conjugated to target proteins by a pathway that is distinct from, but analogous to, the ubiquitin conjugation. Similar to known SUMO E1 enzymes, which are distinct from ubiquitin-specific enzymes (Desterro *et al*., 1997; Johnson and Blobel, 1997), *P. falciparum* E1 consists of a heterodimer of two enzymes, Uba2 and Aos1. The E2 homologue with conjugating activity is the Ubc9, which also acts as the SUMO ligase (Johnson and Blobel, 1997). The pathway is completed with the SUMO proteases, which have the dual function of de-sumoylating the target by cleaving the substrate bound SUMO peptide, as well as to process the mature SUMO by cleaving it to leave the diglycine motif at the terminus for isopeptide bond formation.

### Detection of putative sumoylated proteins in *P. falciparum*

**Fig. 2:**
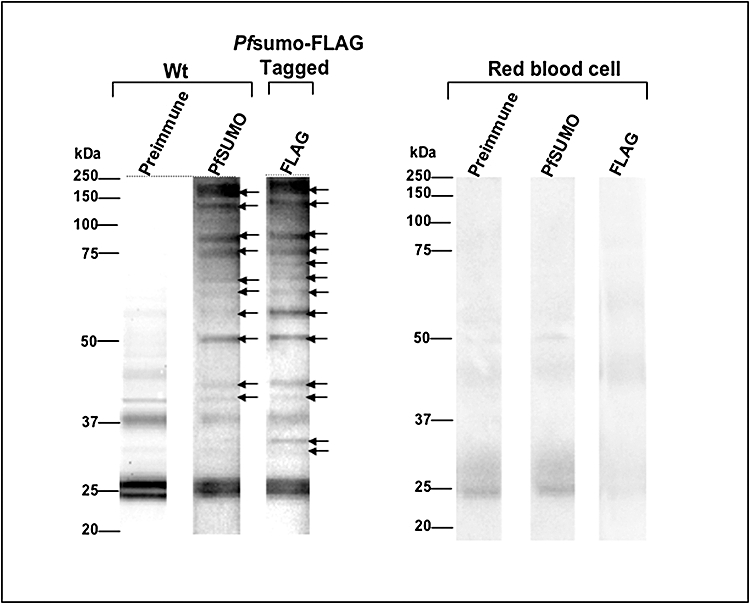
Immunoblot analysis with anti-PfSUMO and anti-FLAG antibodies. Total parasite extracts from mid to late trophozoites (30–32 h post invasion) of 3D7 and 3D7 PfSUMO-FLAG tag expressing parasites were separated by 4–12% SDS-PAGE and immunoblotted with anti-PfSUMO pre-immune, anti-PfSUMO sera and anti-FLAG antibodies. Arrows indicate multiple bands corresponding to probable SUMO substrates identified in 3D7 and PfSUMO tagged parasite extracts by anti-PfSUMO and anti-FLAG antibodies respectively. Extracts from uninfected red blood cells were separated by 4–12% SDS-PAGE and immunoblotted with anti-PfSUMO pre-immune, anti-PfSUMO sera and anti-FLAG antibodies.

Western blot analysis of *P. falciparum* total parasite extracts was performed, revealing several bands corresponding to potentially sumoylated plasmodial proteins (Fig. 2). Anti-PfSUMO guinea pig sera stains a number of different proteins within the range of ∼40 kDa to < 250 kDa in strain 3D7 parasite extracts. The pre-immune sera did not recognize the large majority of the proteins. Similar results were observed in extracts from transfectants bearing endogenously FLAG-tagged PfSUMO (Fig. 2). Mouse anti-FLAG antibody stains an essentially comparable pattern in PfSUMO-FLAG transfectants, although revealing a few more potentially sumoylated proteins, perhaps due to higher sensitivity resulting from tagging. In-depth analysis of the membrane and cytosolic proteome of red blood cell did not reveal any evidence of sumoylated proteins so far (Pasini *et al*., 2006). Nevertheless, to exclude any host-specific sumoylated proteins and confirm specificity of the antibodies, extracts from uninfected red blood cells were also blotted with the above-mentioned antibodies (Fig. 2, right panel). Extracts obtained at specific stages, i.e. the early ring stages, mature trophozoite and schizont stages, revealed differential pattern (data not shown), indicating that the modification targets stage-specific proteins or modifies the same protein differentially in a stage-specific manner.

### Kinetics and localization of sumoylated proteins in infected red blood cells

**Fig. 3:**
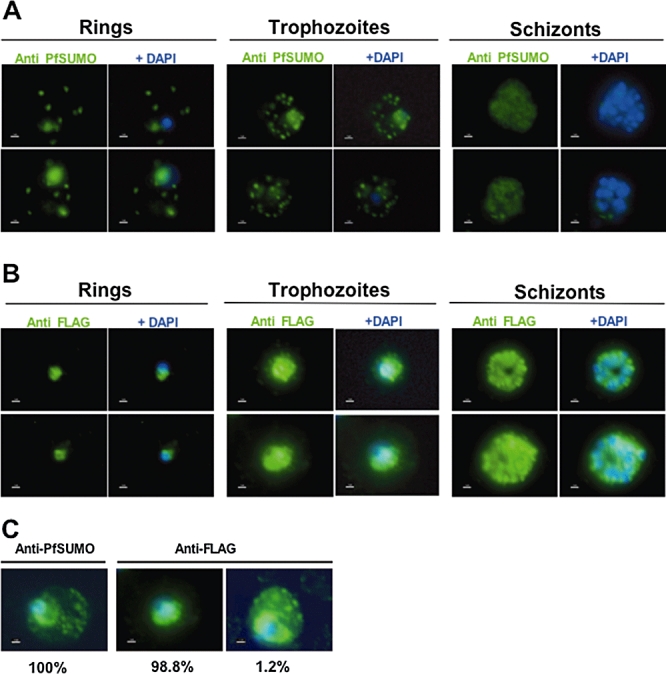
PfSUMO immunofluorescence assays throughout the blood stage cycle. A. Reactivity of anti-PfSUMO sera on wild-type 3D7 strain parasites. B. Reactivity of anti-FLAG (tag) antibody on PfSUMO-FLAG-tagged parasites. Ring stage 10–14 h, trophozoite stage 26–30 h, schizont stage 40–46 h post invasion. C. Comparison of IFA pattern obtained by anti-PfSUMO and anti-FLAG antibodies on PfSUMO-FLAG-tagged parasites (trophozoites). Size bar corresponds to 1 μm. Nuclear staining by DAPI in blue.

To address the question as to where SUMO target proteins localize, we performed immunofluorescence assays (IFAs) on fixed, synchronized wild-type parasites at specific stages with anti-PfSUMO antibodies (Fig. 3A). Staining was observed in all the blood stages with a differing pattern across the stages. Ring stage wild-type 3D7 parasites showed partial nuclear staining along with around 5–6 Maurer's clefts-like vesicular structures in the host cytoplasm. A similar pattern was observed during trophozoite stages but the number of vesicular structures increased (Fig. 3A, middle panel). At schizogony the picture became more dense as diffused staining, mainly throughout the nuclei, was observed (Fig. 3A, right panel). SUMO modification in other systems is known to target mainly nuclear proteins, although many reports implicate SUMO in modifying cytosolic proteins specifically targeting nuclear-cytosolic export. Furthermore, the pattern appeared to be similar to the *P. falciparum* Maurer's clefts, vesicle-like structures that are involved in the trafficking of PfEMP1 and other virulence proteins to the host cell surface. IFAs on PfSUMO-FLAG transfectants yielded similar staining pattern throughout the life cycle when probed with anti-FLAG antibody, with the exception of the Maurer's clefts-like pattern not being detectable in most parasites analysed (Fig. 3B). A minority (approximately 1%) of the PfSUMO-FLAG-infected parasites, when assayed with anti-FLAG antibodies did, however, show the host cytosolic staining (Fig. 3C). This may be explained by altered characteristics of the tagged PfSUMO.

**Fig. 4:**
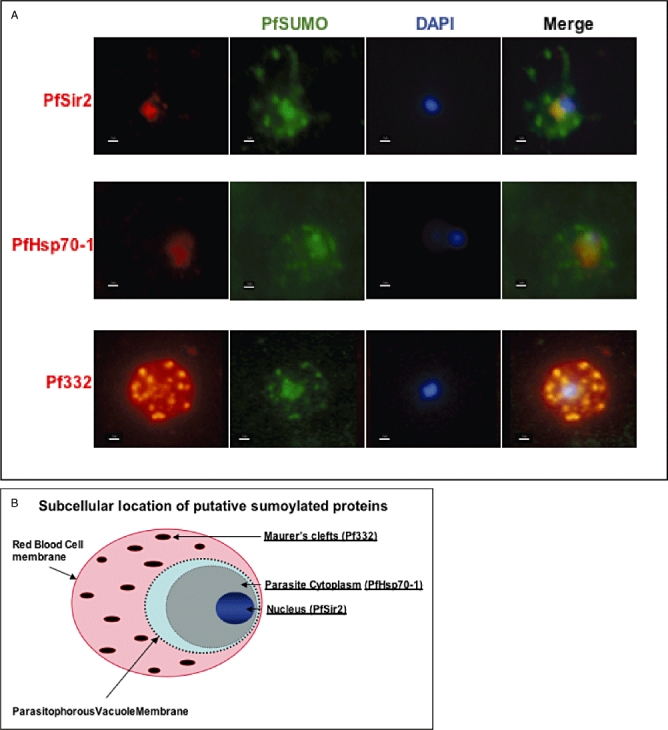
Distinct subcellular localization of PfSUMO conjugated proteins. A. Co-localization assays of PfSUMO antiserum with nuclear marker PfSir2, cytoplasmic marker, PfHsp70-1 and Maurer's clefts marker, Pf332 reveal compartmentalization. Size bar corresponds to 1 μm. B. Schematic. Underlined compartments have been shown to react with anti-PfSUMO antibodies. In the nucleus, PfSir2 has been identified, in this work, as the first putative SUMO target in *P. falciparum*.

However, intrigued by the Maurer's clefts-like staining by PfSUMO sera, we undertook colocalization IFAs with *P. falciparum* markers staining specific parasite compartments and anti-PfSUMO antibodies (Fig. 4). To this end, we used anti-PfSir2 (Freitas-Junior *et al*., 2005), a nuclear marker, and observed partial colocalization of PfSUMO with the peripheral nuclear staining pattern of PfSir2. This demonstrated the nuclear staining by anti-PfSUMO and is consistent with a possible association with PfSir2 (Fig. 4A, top panel). Next we used anti-PfHsp70-1 (Mattei *et al*., 1989) as a cytoplasmic marker and observed a colocalization with PfSUMO (Fig. 4A, middle panel). To further investigate the observed extra parasitic host cytosolic staining, we used a marker that stains the *P. falciparum* Maurer's clefts, mAb51-22 (Hinterberg *et al*., 1994). We observed complete colocalization of the PfSUMO vesicular dots with Maurer's clefts (Fig. 4A, bottom panel). These assays documented the presence of putatively sumoylated proteins in different parasitic and extra parasitic compartments (Fig. 4B).

To test for specificity of the PfSUMO serum antibodies, we performed peptide competition assays and subsequent immunofluorescence. The specific staining pattern in the parasite and host cell could be inhibited in a dose-dependent manner conferring that the IF staining is specific (Fig. S2). IFAs were also carried out with non-fixed, air-dried parasites and identical results obtained (data not shown).

### Identification of PfSir2 as a SUMO substrate

**Fig. 5:**
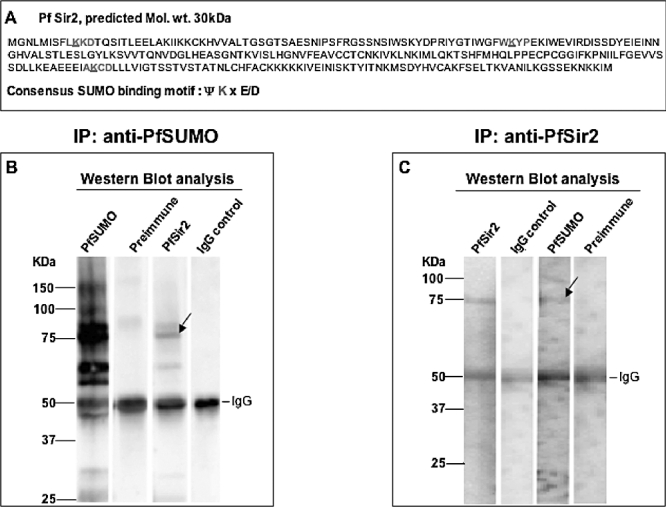
Immunoblot analysis of immunoprecipitated PfSir2 and sumoylated proteins. A. Sequence of PfSir2 obtained from PlasmoDB tested on SUMOplot to scan for potential SUMO acceptor motifs (ψ K x E/D, with ψ – a hydrophobic residue, K – acceptor lysine to which the di-glycine motif of the SUMO peptide is attached, x – any amino acid followed by a Glutamic acid or Aspartic acid). Coloured amino acids depict probable motifs with red depicting high probability motif and blue lower probability, critical lysine residue with the motif is underlined. B. Proteins immunoprecipitated from nuclear extracts with anti-PfSUMO sera were run on a 4–12% gradient gel and immunoblotted with anti-PfSUMO sera and pre-immune sera as positive controls (first two lanes), anti-PfSir2 (third lane) and anti-IgG (fourth lane). Arrow marks corresponding PfSir2 band(s) observed in anti-PfSUMO immunoprecipitated protein extract. C. Proteins immunoprecipitated with anti-PfSir2 sera were run on a 4–12% gradient gel and immunoblotted with anti-PfSir2 sera and anti-IgG as positive controls (first two lanes), anti-PfSUMO (third lane) and anti-PfSUMO pre-immune sera (fourth lane). Arrow marks corresponding sumoylated fraction observed in anti-PfSir2 immunoprecipitated protein(s).

PfSir2 is a nuclear plasmodial protein that is implicated in control of *var* gene transcription (Duraisingh *et al*., 2005; Freitas-Junior *et al*., 2005). During characterization of this protein we observed a discrepancy from the predicted molecular weight of 30 kDa and instead observed a major band of approximately 72 kDa on immunoblotting with anti-PfSir2 sera (Freitas-Junior *et al*., 2005). We questioned whether this shift in size could be due to a post-translational modification like sumoylation. On scanning the PfSir2 for probable sumoylation motifs (Rodriguez *et al*., 2001), a consensus sequence of four amino acids, most widely validated as SUMO binding motif (ψ K x E/D), we found three motifs, one with high probability and two with low probability that were potential SUMO binding sites (http://www.abgent.com/doc/sumoplot, Fig. 5A). Following this we carried out immunoprecipitation (IP) assays with anti-PfSir2 and anti-PfSUMO antibodies and probed the precipitated fractions with anti-PfSUMO and anti-PfSir2 sera respectively (Fig. 5B and C). The fraction immunoprecipitated with anti-PfSUMO (Fig. 5B) recognized a faint yet distinct band at around 72 kDa on being probed with anti-PfSir2 along with a fainter fraction at around 60 kDa. The same fraction when blotted with PfSUMO as positive control recognized many proteins, as can be deduced from the multiple bands obtained. The control antibodies, namely PfSUMO pre-immune and IgG (as control for rabbit sera of PfSir2), did not reveal any of the above-mentioned specific bands. We repeated this using PfSir2 for immunoprecipitation and probing with PfSUMO and obtained corresponding results (Fig. 5C), thus ascertaining modification of PfSir2.

### Identification of sumoylated candidate proteins by liquid chromatography-tandem mass spectrometry LC-MS/MS (LTQ)

Sumoylation is a global modification with widespread targets and far-reaching effects. We were interested in generating a first list of putative SUMO targets in *P. falciparum* to gain insight into the cellular processes affected by this modification. Taking advantage of the FLAG-tagged PfSUMO strain, we conducted a two-step purification of SUMO target proteins. IPs were carried out, first with monoclonal anti-FLAG antibody followed by anti-PfSUMO sera, thus increasing the stringency of the purification. Accurate mass (MS) and sequence information (MS/MS) was obtained by standard in-gel trypsin digestion of bands specific only to PfSUMO sera and treatment of resulting eluates in the mass spectrometer. Information acquired was used to identify the peptides against the *Plasmodium* database using SEQUEST algorithm. A list of corresponding proteins was generated based on the analysis (Table 1). Although a number of hits were obtained using stringent filtering criteria, only a fraction was identified by two or more peptides generating more confidence than those identified by single peptides. This is not surprising, considering the reported low abundance of sumoylated proteins at a given time in analogous systems. It is noteworthy though that among all the substrates recognized, a good number had orthologues known to be sumoylated individually and/or as part of a complex in other model systems (marked by asterisk ‘*’ in Table 1). All the identified proteins harbour potential sumoylation motifs (data not shown), though due to the limitation of the mass spec technique, the exact residue(s) could not be identified.

**Table 1:** Putative SUMO substrates identified by LC-MS/MS analysis of *Plasmodium falciparum* proteins.

Gene ID^a^	Protein name	Putative function	Location	No. of peptides
Proteins identified as sumoylation substrates by two or more peptides				
PFD0415c	Hypothetical Protein	ND	ND	6
	Hypothetical Protein	ND	ND	6
PFC0140c	N-ethylmaleimide-sensitive fusion protein, putative	Vesicular trafficking	IE cytoplasmic vesicular structures	6
PF14_0326*	Dynein-related AAA-type ATPase	Invasion	Merozoite	5
PFL1525c*	Pre-mRNA splicing factor RNA helicase, putative	Splicing	ND	3
PF14_0314	Chromatin assembly factor 1 p55 subunit	Transcription factor	ND	2
PF14_0348	ATP-dependent Clp protease proteolytic subunit, putative	ND	ND	2
PF10_0161	PHIST domain protein	ND	ND	2
Proteins identified as sumoylation substrates by single peptide				
PF11_0061*	Histone H4	Chromatin structure	ND	
PF14_0334	NAD(P)H-dependent glutamate synthase, putative	Glutamate metabolism	ND	
PFD0735c	Hypothetical Protein	ND	ND	
PF07_0030	Heat shock protein 86 family	Heat shock protein	ND	
PFL0120c*	Cyclophilin, putative	Intracellular protein folding and signal transduction	ND	
PF11_0381	Subtilisin-like protease2	Invasion	Micronemes	
PF10_0055	Hypothetical Protein	ND	ND	
PFE1120w	Hypothetical Protein	ND	ND	
PF11_0404*	Transcription factor with AP2 domain(s)	Transcription factor	ND	
PF14_0067	LCCL domain-containing protein CCP3	ND	ND	
PF14_0713	RNA-binding protein/splicing factor	RNA binding/splicing	ND	
MAL7P1.68	Zinc finger containing ERF2 subunit of a palmitoyltransferase	Palmitoylation	ND	
MAL8P1.7	DNA-directed RNA polymerase II second largest subunit, putative	Transcription	ND	
PFF0975c	Conserved plasmodium protein	ND	ND	
PFF1440w*	SETdomain protein	Chromatin structure	ND	
PF10_0368*	Dynamin protein	Trafficking/organelle fission/ Cytoplasm, ER -Golgi, Apicoplast		

a http://plasmodb.org

* Orthologues known to be directly sumoylated and/or part of sumoylated complex.

ND, not determined; IE, infected erythrocyte.

## Discussion

In this work we present, for the first time, experimental evidence that *P. falciparum* utilizes SUMO conjugation to modify blood stage proteins. Antibodies raised against a *P. falciparum* SUMO peptide orthologue as well as against the epitope used to tag the PfSUMO detect, by Western blot, more than a dozen parasite proteins expressed during the intracellular blood stage cycle. Many proteins seen in total parasite extracts are also detected in nuclear extracts (Figs 2A and 5A), indicating that the majority of sumoylated proteins reside in the nucleus. Unlike ubiquitination, SUMO conjugated proteins are not marked for degradation, but rather have a number of eclectic functions in eukaryotic organisms such as in protein–DNA interactions (Goodson *et al*., 2001; Hong *et al*., 2001), regulation of transcription factor and chromatin-remodelling protein(s) (Gill, 2003) among many other biological processes. A knowledge of the proteins targeted by this modification is thus of utmost importance in deciphering its impact on the biology of the organism. To this end, we have also generated the first list of candidate-sumoylated proteins in plasmodium by mass spectrometry approach. Some of these are of particular interest for the parasite biology (Table 1) and may be pursued for further investigation. Given the limiting amount of plasmodial extracts obtainable as compared with other model organisms (i.e. yeast, mammalian) as also the low abundance of enriched SUMO conjugates present at a given time, identification of sumoylated targets is technically challenging and requires an upscaling of the initial starting material and more sensitive instrumentation.

The staining of anti-PfSUMO with Maurer's clefts indicates that the parasite may traffic SUMO-conjugated proteins outside the conventional intracellular compartments into the erythrocyte cytoplasm (see Fig. 4B). The robust appearance of an NSF fusion protein in the mass spec analysis complements this observation (Table 1). This protein is known to appear as cytoplasmic vesicular structures with putative role in vesicular trafficking (Hayashi *et al*., 2001). Combining these observations, it is tempting to speculate that PfSUMO may take part in the regulation of virulence factor trafficking to the infected erythrocyte (IE) surface, as has been described for another post-translational modification, the phosphorylation of Maurer's clefts proteins by parasite exported kinases (Droucheau *et al*., 2004; Nunes *et al*., 2007). Our efforts to identify sumoylated proteins in purified ghosts of IE failed using our anti-PfSUMO antibodies (data not shown). This may be due to the low abundance of proteins that carry this modification. In FLAG-tagged parasites, only a minor fraction of the IE cytoplasm was stained, indicating that the pathway leading to the Maurer's clefts trafficking may be disturbed by the presence of the FLAG tag.

Having observed the unusual molecular weight of the nuclear protein PfSir2 in Western blot in nuclear extracts, we speculated that it may be a substrate for SUMO ligase(s). The open reading frame predicts a protein of approximately 30 kDa and the apparent MW is 72 kDa using a number of different anti-PfSir2 antibodies (Freitas-Junior *et al*., 2005). Given that three putative sumoylation sites are found in the PfSir2 sequence (Fig. 5A), full sumoylation would predict a molecule of approximately 70 kDa. Our data strongly support SUMO conjugation causing the increase in molecular weight of PfSir2. The fainter band at approximately 52 kDa (Fig. 5B) could be either a differentially spliced form or a degradation product. PfSir2 is known to be associated with the nuclear periphery with telomeres and nucleolus. We hypothesize that PfSir2 sumoylation determines the nuclear location of PfSir2. Mutant PfSir2 revealed in *P. falciparum* that this protein is involved in a number of different functions, such as gene silencing of a subgroup of telomere-adjacent *var* genes (Duraisingh *et al*., 2005; Freitas-Junior *et al*., 2005) and telomere length control (A. Scherf, unpubl. data). Work to investigate whether mutating the three putative SUMO conjugation sites may lead to differential subcellular location of PfSir2, or alter *var* gene de-repression and telomere length, is underway. The yeast Sir2 is not sumoylated (Denison *et al*., 2005). However, other HDACs have been shown to be sumoylated (David *et al*., 2002; Cheng *et al*., 2004) with altered biological activity in terms of levels of protein acetylation and/or gene repression. Interestingly, very recent studies show SIRT1, the closest mammalian homologue of yeast Sir2, to be sumoylated thereby increasing its deacetylation activity (Yang *et al*., 2007).

Histone sumoylation in *S. cerevisiae* has been recently observed, implicating it as an alternative mechanism akin to other chromatin modifications, such as methylation and acetylation (Nathan *et al*., 2006). We investigated whether *P. falciparum* histones carry SUMO marks. Histone H4 specifically came up as a SUMO substrate in our mass spec analysis (Table 1). Using semi-purified histones and reacting with anti-PfSUMO sera, we observed a faint band migrating higher than the predicted molecular weight of histones (this work, unpublished data). Also, this observation is compatible with a minor fraction of histones being conjugated to SUMO and argues for a possible role of sumoylation as a negative regulator in malaria parasites. Interestingly, the H2AZ homologue seemed to accumulate as many as six potential SUMO motifs in the N-terminal region (H2AZ – 6 sites – K11, K15, K25, K30, K38, K153; H2A – 4 sites K6, K21, K36 and K124), outnumbering those present in yeast H2AZ and H2A. Similar tandem array of potential sumoylation sites in the N-terminal region were also seen on other apicomplexans, e.g. the *T. gondi* H2AZ (with seven motifs in the N-terminal). Given the exposure of the N-terminal region of histones to enzymatic modifications, it points to a possible differential utilization of the same lysine residues for different modifications.

Stabilizing proteins by competing with ubiquitin (Desterro *et al*., 1998) and regulation of binding topromoter DNA (Boyer-Guittaut *et al*., 2005) were the only mechanisms known to be associated with SUMO modification of transcription factors, strictly limiting it to target-SUMO covalent conjugation. Recently, though SUMO has been suggested to have a transcriptionally repressive role, mainly by promoting non-covalent interactions with co-repressors. Multitude of chromatin (histone) modifying enzymes, including deacetylases, methyltranferases and demethylases, are proposed to be among the many transcriptional regulators that interact non-covalently with SUMO to bring about repression (Yang *et al*., 2003; Rosendorff *et al*., 2006). In line with this, a number of putative sumoylated proteins identified in our mass spec study are potential transcription and chromatin regulators and orthologues of some of those identified are known SUMO targets (Table 1). The proteins detected in our analysis are probably only the most dominant ones and represent only ‘the tip of the iceberg’. Nonetheless, identification of SUMO – interacting domains on these and other potential candidates will be of utmost importance. Moreover, the physical association of SUMO with large multiprotein complexes like nuclear lamin proteins, PML nuclear bodies and centromeres may suggest SUMO in maintaining stability of multiprotein complexes (Zhong *et al*., 2000; Seeler *et al*., 2001), an interesting avenue to explore in a system like *Plasmodium* where multiprotein complexes are known to work in tandem for important processes like trafficking and silencing, etc.

In summary, despite the growing knowledge on the importance of this relatively novel modification, little is known about its precise biological impact/outcome even in extensively studied model systems, and none in apicomplexan before this study. The list of newly discovered SUMO substrates is expanding only recently in other model systems whereas none existed so far for plasmodium before this study. No unifying role could be so far associated with sumoylation in eukaryotes and it appears that each studied class of protein adds a new function to the long list of described biological roles (Seeler *et al*., 2007; Xhemalce *et al*., 2007). Our work on malaria parasites supports this notion, as we observe sumoylation of the telomere-associated protein PfSir2, involved in gene silencing, and we present evidence of a putative SUMO substrate that is exported from the parasite into the host cytoplasm via Maurer's clefts. Both observations have not been previously associated with SUMO and support the notion in malaria parasites of ‘The usual suspect in a new scenario’. Obviously, a first logical step would be to do an in-depth and detailed proteomic profile of the same to identify more SUMO substrates in blood stage parasites of *P. falciparum* and identify their critical role in the biology of the parasite such as immune escape, invasion process and gene regulation. Both *Plasmodium*-specific anti-PfSUMO antibody and the FLAG-tagged SUMO strain generated in our work provide a valuable tool to explore the biological impact of this modification in the pathogen. The list of putative plasmodial SUMO substrates further provides direction to this understanding.

## Experimental procedures

### P. falciparum*cultures*

*Plasmodium falciparum* blood stage parasites from the 3D7 strain were cultured using modifications to the method described by Trager and Jensen (1976). Briefly, parasites were grown in O+ human erythrocytes in RPMI 1640 medium containing l-glutamine (Invitrogen) supplemented with 5% v/v human AB serum (PAA Laboratories GmbH) and 5% v/v Albumax II (Invitrogen) in a gas environment of 5% CO_2_, 1% O_2_ and 94% N_2_. Cultures were synchronized with two consecutive 5% sorbitol (Sigma) treatments (Lambros and Vanderberg, 1979). For kinetic IFAs the parasites were synchronized within a window of approximately 6 h, monitored by Giemsa staining.

### Bioinformatic analysis

Genes coding for putative ubiquitin-like proteins in *P. falciparum* were obtained both from PlasmoDB (http://www.plasmodb.org) and GeneDB (http://www.genedb.org/genedb/malaria/). To identify the plasmodial SUMO gene (PfSUMO), previously characterized yeast SUMO/SMT3 orthologues (YDR510W) were obtained from NCBI (http://www.ncbi.nlm.nih.gov/BLAST/) and used as query on TBLASTN. Multiple sequence alignments were constructed using CLUSTALW algorithm using the BLOSUM series of similarity matrix with gap opening and extension penalties of 10 and 0.1 respectively.

### Antibodies

To obtain polyclonal antibody serum against PFE0285c (*Pf*SUMO1), we used the Eurogentec standard protocols (http://www.eurogentec.com) for immunizing guinea pigs and mice. Each animal was immunized with a combination of two synthetic peptides. The peptide sequences are as follows: H2N-[+C]QGEHIQVKVRSPDGA-CONH2 and H2N-[ + C]YDGDRIHGDNTPEQL. blast search using individual peptide sequence(s) against the *P. falciparum* database did not yield any significant hits. Anti-FLAG mouse monoclonal antibody was obtained from Sigma. Co-localization assays were carried out with antibodies directed against specific markers: nuclear marker, PfSir2 (Freitas-Junior *et al*., 2005), parasite cytoplasmic marker, PfHsp70-1 (Mattei *et al*., 1989) and Maurer's clefts marker, mAb51-22 (anti-Pf332) (Hinterberg *et al*., 1994).

### Plasmid construct and parasite transfection

PfSUMO gene was amplified using Expand High Fidelity PCR System (Roche) with primers containing restriction sites for the enzymes XhoI and AflII, forward primer 5′-CCG**CTCGAG**ATGGGAGATGACGATTCAGC-3′, reverse primer 5′-AAAAAA**CTTAAG**TTAGAAACTTCCTCCTGTTTGCTGAACC-3′ (enzyme restriction sites in bold) and cloned in frame at the C terminal of 1× FLAG tag previously cloned into the backbone plasmid vector pLN-ENR-GFP (Nkrumah *et al*., 2006). 3D7 ring stage parasites were transfected by electroporation and drug selected using bacterial integrase mediated transfection system as previously described (Nkrumah *et al*., 2006) to obtain chromosomal integration of the tagged construct.

### Immunofluorescence microscopy

Synchronized IEs (ring stage 10–14 h, trophozoite stage 26–30 h, schizont stage 40–46 h) were washed in phosphate-buffered saline (PBS) (0.15 M NaCl and 10 mM sodium phosphate, pH 7.2). Cell pellets were re-suspended in 5 vols of PBS and a monolayer was set onto microscope slides. Parasites were air-dried and fixed for 10 min at room temperature in 4% paraformaldehyde. Slides were blocked for 20 min in 0.1% bovine serum albumin (BSA) and incubated with the primary antibody diluted in 0.1% BSA for 45 min at room temperature. After washing, cells were incubated for 30 min with a secondary antibody conjugated with appropriate fluorochrome (Alexa Fluor 488, Alexa Fluor 568, Molecular Probes) in a 1:500 dilution. Slides were washed thoroughly in PBS and mounted in Vectashield anti-fading containing 4-6-diamidino-2-phenylindole (DAPI) for nuclear staining (Vector Laboratories). Images were captured using a Nikon Elipse 80i optical microscope.

Anti-PfSUMO sera were pre-absorbed with lysed erythrocytes before incubation with the IEs. The final antibody dilutions were 1:200 for both PfSUMO sera and pre-immune, 1:500 for anti-FLAG, 1:100 for anti-PfSir2, 1:500 for anti-PfHsp70-1 and 1:400 for anti-mAb51-22 for colocalization assays.

Peptide competition assays were performed to test the specificity of the anti-PfSUMO sera. Increasing concentrations of the specific and unspecific peptides, respectively (ranging from 3 μg of respective peptide per ml to 3000 μg per ml), were added to the serum and incubated overnight in a rotating wheel at 4°C. The serum was subsequently used to carry out IFA as described above.

### Western blot analysis

Equal amounts of parasite protein extracts from 3D7 strain and PfSUMO-FLAG transfected strain were separated by 4–12% SDS-PAGE on gradient gels (Invitrogen) and transferred onto nitrocellulose membranes. Membranes were blocked at room temperature for 1 h in PBS, 0.05% Tween-20 and 5% non-fat milk and probed with antibody against PfSUMO and pre-immune sera overnight at 4°C. Secondary antibodies conjugated to horseradish peroxidase (Pierce) were incubated for 1 h at room temperature and developed with SuperSignal West Pico Chemiluminescent Substrate (Pierce) according to the manufacturer's instructions. Precision Plus Pre-stained molecular weight marker was obtained from Bio-Rad.

### Immunoprecipitation assays

Briefly, ∼4 × 10^7^ parasites (trophozoite stage 26–30 h post invasion) were isolated from IE by saponin lysis. Parasites were re-suspended in Lysis buffer [10 mM Hepes (pH 7.9), 10 mM KCl, 0.1 mM EDTA, 0.1 mM EGTA, 1 mM DTT, 0.25% NP-40] containing a mixture of protease inhibitors (Roche) and set on ice for 30–60 min. Parasites were then lysed by 100–300 strokes in a homogenizer, the nuclei recovered by centrifugation, re-suspended in 100 ml of sonication buffer and then incubated on ice for 15 min followed by sonication with a Bioruptor Sonicator according to manufacturer's instructions. After sonication-insoluble material was removed by centrifugation, lysates were pre-cleared by incubation with protein A sepharose CL-4B (Amersham) for 1 h. The extracts were incubated overnight at 4°C with 15 μl each of PfSUMO antisera and the pre-immune serum, anti-PfSir2 and anti-IgG. Immunoprecipitates were collected with protein A sepharose, and washed once each with low-salt wash buffer [0.1% SDS, 1% Triton X-100, 2 mM EDTA, 20 mM Tris-HCl (pH 8.1), 150 mM NaCl, protease inhibitors], High-salt wash buffer [0.1% SDS, 1% Triton X-100, 2 mM EDTA, 20 mM Tris-HCl (pH 8.1), 500 mM NaCl, protease inhibitors] and LiCl-immune complex wash buffer (0.25 M LiCl, 1% NP-40, 1% deoxycholate, 1 mM EDTA, 10 mM Tris-HCl [pH 8.1], protease inhibitors) followed by a wash in TE buffer. The immunoprecipitated proteins were eluted from the sepharose beads by addition of Lamelli buffer and boiling for 10 min at 95°C. The eluates were separated on 4–12% SDS gels and blotted with appropriate antibodies as described above.

### Mass spectrometry analysis

Two-step IPs were performed, as above, on extracts from asynchronous blood stage cultures of PfSUMO-FLAG transfected strain. Monoclonal anti-FLAG antibody was used in the first step IP and eluate was subjected to second IP with anti-PfSUMO and pre-immune sera respectively. After standard in-gel trypsin digestion, samples were subjected to MS/MS spectrometry using a LTQ ion trap mass spectrometer. The collection of resulting spectra was then searched against a plasmodium database (PlasmoDB) using the SEQUEST algorithm. Pepetides identified were subjected to stringent search criteria with Xcorr values greater than 1.9, 2.2, 3.75 and 4.5 for 1, 2, 3 and 4 spectra, respectively, and Delta CN > 0.1.

## Supplementary material

The following supplementary material is available for this article online:

Fig. S1 PfSUMO versus ubiquitin alignment. CLUSTALW algorithm was used to ascertain PfSUMO homology to known ubiquitin and Ubl orthologues. Multiple alignment between human ubiquitin and Nedd8, S. cerevisiae Ubiquitin and Nedd8-like RUB1 and S. pombe Nedd8 was done. Dark shading represents residues identical in all sequences. Light shading, residues similar between respective sequnces. Box, conserved Lys 48 and Lys 63, which serve as common sites of ubiquitination polymerization, which are missing in PfSUMO sequence, as pointed with arrow(s).

Fig. S2 PfSUMO peptide competition assay. IFA after competing PfSUMO antisera with PfSUMO peptide (A) and unspecific peptide (B). Horizontal grey bar above IFA panel depicts increasing amount of specific and unspecific peptide used, respectively, to compete the antisera (3–3000 mg ml-1).

This material is available as part of the online article from: http://www.blackwell-synergy.com/doi/abs/10.1111/

j.1462-5822.2008.01183.x
