# Supplementary figures and images for: Identification of a novel post-translational modification in Plasmodium falciparum: protein sumoylation in different cellular compartments
Source: Cell Microbiol. 2008 Oct;10(10):1999–2011. doi: 10.1111/j.1462-5822.2008.01183.x (PMC2613257; doi:10.1111/j.1462-5822.2008.01183.x)

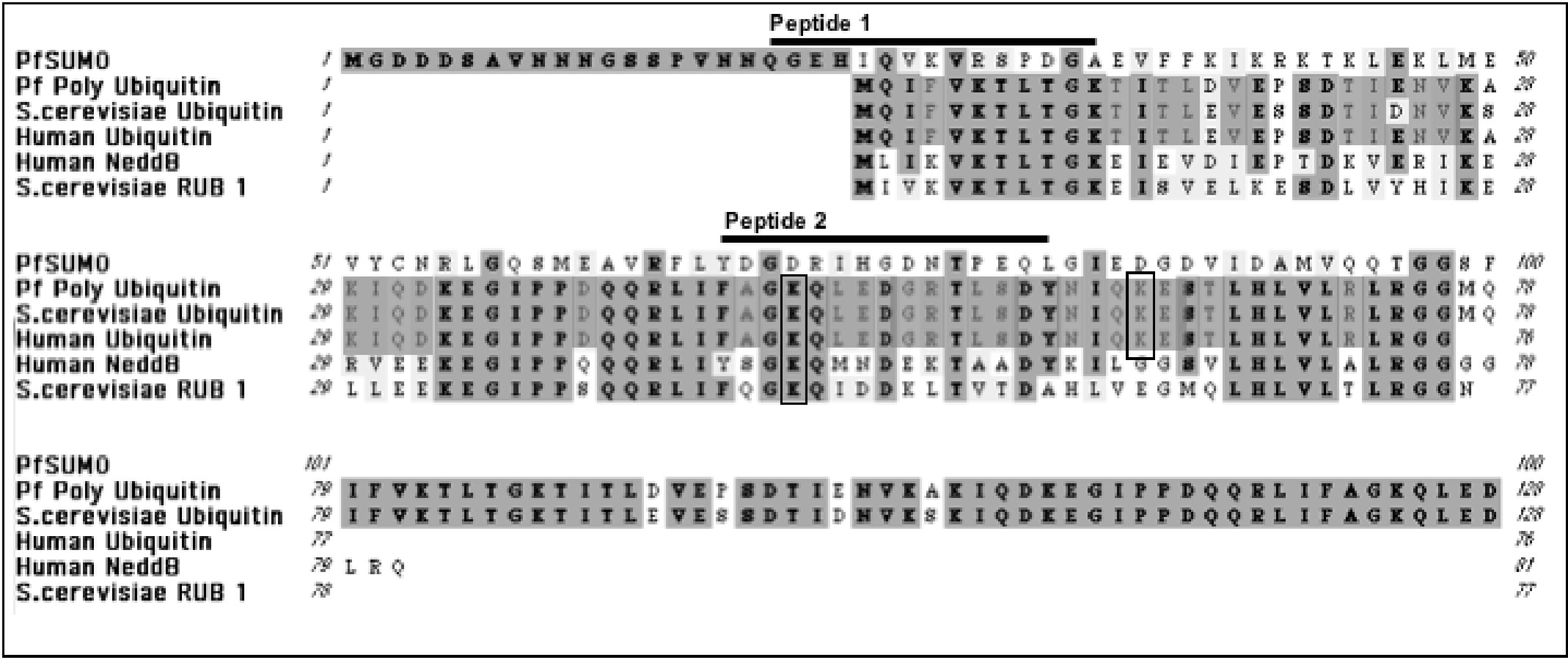

Supplement: Fig. S1 — PfSUMO versus ubiquitin alignment. CLUSTALW algorithm was used to ascertain PfSUMO homology to known ubiquitin and Ubl orthologues. Multiple alignment between human ubiquitin and Nedd8, S. cerevisiae Ubiquitin and Nedd8-like RUB1 and S. pombe Nedd8 was done. Dark shading represents residues identical in all sequences. Light shading, residues similar between respective sequnces. Box, conserved Lys 48 and Lys 63, which serve as common sites of ubiquitination polymerization, which are missing in PfSUMO sequence, as pointed with arrow(s). [file cmi0010-1999-sd1.tif]
